# Aging, Osteocytes, and Mechanotransduction

**DOI:** 10.1007/s11914-017-0402-z

**Published:** 2017-09-11

**Authors:** Haniyeh Hemmatian, Astrid D. Bakker, Jenneke Klein-Nulend, G. Harry van Lenthe

**Affiliations:** 1 Biomechanics Section, Department of Mechanical Engineering, KU Leuven, Celestijnenlaan 300c, 3001 Leuven, Belgium; 2 Department of Oral Cell Biology, Academic Centre for Dentistry Amsterdam (ACTA), University of Amsterdam and Vrije Universiteit Amsterdam, Amsterdam Movement Sciences, Amsterdam, The Netherlands

**Keywords:** Osteocyte lacuna, Aging, Mechanotransduction, Bone mechanobiological response

## Abstract

**Purpose of Review:**

The bone is able to adapt its structure to mechanical signals via the bone remodeling process governed by mechanosensitive osteocytes. With aging, an imbalance in bone remodeling results in osteoporosis. In this review, we hypothesized that changes in lacunar morphology underlie the decreased bone mechanoresponsiveness to mechanical loading with aging.

**Recent Findings:**

Several studies have reported considerable variations in the shape of osteocytes and their lacunae with aging. Since osteocytes can sense matrix strain directly via their cell bodies, the variations in osteocyte morphology may cause changes in osteocyte mechanosensitivity. As a consequence, the load-adaptive response of osteocytes may change with aging, even when mechanical loading would remain unchanged.

**Summary:**

Though extensive quantitative data is lacking, evidence exists that the osteocyte lacunae are becoming smaller and more spherical with aging. Future dedicated studies might reveal whether these changes would affect osteocyte mechanosensation and the subsequent biological response, and whether this is (one of) the pathways involved in age-related bone loss.

## Introduction

Osteoporosis is a prevailing skeletal disease of aging [1••]. It is defined by low bone mass and associated with deterioration of the bone microarchitecture, leading to reduced bone strength and increased risk of fragility fractures. Osteoporosis is an economic as well as health burden resulting in 8.9 million osteoporotic fractures worldwide annually [2]. Hence, there is a strong socio-economic need to reduce the number of fractures. A better understanding of the biological mechanisms underlying bone loss with aging is crucial to reach this objective.

The skeleton is a dynamic organ with the capacity to adapt itself to its mechanical environment [3]. Bone adaptation to mechanical loading typically results in the formation of a bone structure that provides an appropriate resistance to fractures while using a small amount of material. The process of bone adaptation is controlled by mechanosensitive osteocytes. Osteocytes sense mechanical signals placed upon the bone, and consequently orchestrate the activity and recruitment of osteoblasts and/or osteoclasts by producing a multitude of signaling molecules (for extensive reviews, see Klein-Nulend et al., 2013 [4••, 5••]). Bone mass is determined by the overall metabolic action of the osteoblasts and osteoclasts, while local bone architecture results from local osteoblast and/or osteoclast recruitment by osteocytes [1••].

Age-related fragility fractures could reflect a deficit in bone mass and/or structural integrity, the main determinants of bone strength. A deficit in bone mass results in part from sex hormone insufficiency, in particular, reduced estrogen levels in postmenopausal women, leading to an overall negative balance between bone resorption and formation rate. An impaired bone architecture may result from a reduced osteocyte ability to control local osteoblast and/or osteoclast recruitment. Indeed, there is ample evidence linking diminished osteocyte sensitivity to age-related bone loss [6, 7]. Thus, the combination of estrogen deficiency and impaired mechanosensitivity of osteocytes might create a major risk for osteoporotic bone fractures [1••].

The ability of osteocytes to sense and respond to mechanical stimuli depends on many factors, such as the shape of the osteocyte cell bodies, number and length of the cell processes, structure of the cytoskeleton, and presence of primary cilia. One intriguing factor is osteocyte lacunar shape, which has been hypothesized to affect the transduction of strain on the whole bone to the direct osteocyte microenvironment [8]. It has been shown that considerable variations in the shape of osteocytes and their lacunae exist and that these variations may depend on anatomical location and the age of the bone [9•, 10•, 11••]. Considering that a proper response of osteocytes to mechanical stimuli is highly important to maintain bone strength, we will address in this review the potential changes in osteocyte mechanosensivity with aging, and answer the question whether changes in lacunar morphology could underlie the alterations in the bone adaptive response seen with aging. We will also discuss novel methods for three-dimensional (3D) visualization and quantification of the lacunar network. In this review, we consider aging the cause of alterations in bone structure at both the micro and macrolevel. Furthermore, we consider osteocytes as mechanosensors that can modify their microenvironment, thereby affecting their mechanosensation and subsequent bone mechanobiological response.

## The Role of Osteocytes in Bone Turnover

The osteocytes are considered to be the cells responsible for sensing mechanical signals on the bones and consequently orchestrating the activity of osteoblasts and osteoclasts (reviewed in Klein-Nulend et al., 2012 [5••]). The osteocyte cell bodies are embedded within the calcified bone matrix and reside in small cavities named lacunae. The cell bodies are interconnected through long dendritic cell extensions (50–60 per cell) which reside in small canals named canaliculi. The lacunae together with the canaliculi form the lacuno-canalicular network (LCN). The large surface area of osteocytes and their long processes allow fast transduction of signals.

The osteocytes are highly mechanosensitive cells and capable of directly influencing the bone-resorbing osteoclasts as well as the bone-forming osteoblasts. Mechanical stimulation of the osteocytes causes changes in their metabolic activity, i.e., they start to produce signaling molecules like Wnts, bone morphogenetic proteins (BMPs), nitric oxide (NO), and prostaglandin E2 (PGE2), thereby adjusting the differentiation, recruitment, and action of osteoblasts and osteoclasts, clearly pointing towards a mechanosensory function of the osteocytes [12–14]. Furthermore, there is substantial evidence that osteocytes are capable of changing their enzyme activity and RNA synthesis in the intact bone quickly after mechanical loading [15–17]. Ablating 80% of the osteocytes prevents the bone loss typically seen after unloading [18]. Thus, osteocytes seem to act as crucial regulators of osteoclastic resorption of the bone [13, 18, 19]. The production of RANKL by osteocytes regulates bone resorption, indicating the essential role of osteocytes in steering osteoclast activity [20, 21].

The mechanisms by which osteocytes sense the mechanical loading, and which mechanical signal is actually being sensed, are poorly understood. The cytoskeleton likely plays a key role. It consists of a composite gel-like material of actin, microtubules, intermediate filaments, and their cross-linkers, and forms the scaffold determining cellular shape and stiffness [22]. Integrins anchor to the extracellular matrix and mechanically link the cell exterior to the cytoskeleton, forming transmembrane complex structures. These complexes are often clustered in focal adhesions, and likely function as mechanotransducers [23, 24]. The importance of anchoring mechanotransduction complexes that connect the extracellular matrix to the cytoskeleton predicts that the osteocyte cytoskeleton plays a key role in osteocyte mechanotransduction (reviewed in Klein-Nulend et al., 2012 [5••]). The cytoskeleton also determines the material and mechanical properties of the cells (resistance to shear or compression), enables cell migration, and is important for the transduction of intracellular molecules [5••].

There are several potential ways for osteocytes to sense mechanical loading [4••, 5••]. First, bone matrix deformations resulting from mechanical loads placed on the bone cause interstitial fluid flow through the canaliculi along the osteocyte cell processes. Evidence for the occurrence of fluid flow in the bone has been provided by a study in mouse tibia [25]. The interstitial fluid flow “amplifies” tissue-level strains and gives rise to the secretion of signals by the osteocytes, which modulate the activity of osteoblasts and osteoclasts, resulting in an adaptive response to mechanical loading [25–29]. Second, the hydraulic pressure induced by loading could be another mechanism to activate osteocytes. It has been shown that a cyclic hydraulic pressure of 68 kPa results in the production of signaling molecules in murine MLO-Y4 osteocyte-like cells, and a pressure as low as 13 kPa induces prostaglandin production by primary osteocytes in chicken calvariae [28, 30]. A third mechanism for sensing mechanical loading may be a direct response to matrix strains [31]. A substrate strain of 3400 microstrain is sufficient to increase the production of signaling molecule by osteoblasts [32]. Since osteocytes are more mechanosensitive than osteoblasts [28], one may expect that osteocytes would respond to lower strain levels. The inhomogeneities in the bone microstructure due to the osteocyte lacunar network can locally amplify the matrix strain to a magnitude that is sufficient to directly activate the osteocyte cell bodies [31].

From a mechanical point of view, it is to be expected that the three strain-sensation mechanisms are affected by lacunar shape, because this would affect the (shear) stresses applied to osteocytes, and lacunar shape could affect the strain amplification around the cell body. Furthermore, the magnitude of the osteocyte signal is likely related to the number of osteocytes contributing to the signal, hence, would be related to the number of lacunae. In order to elaborate these effects, an accurate quantification of the osteocytes and the LCN is essential. In the following sections, we will summarize different imaging techniques of the LCN.

## Visualization of Osteocyte Lacunar Network

Direct quantitative analyses of osteocytes are extremely challenging, because preservation and 3D-visualization of osteocytes are difficult. In addition, the osteocytes are embedded in a stiff and strong bone, complicating the analyses further. Therefore, the osteocyte lacuna is often used as a proxy. Several techniques have been introduced to visualize and quantify osteocyte lacunar network including histological methods, light microscopy (LM), confocal laser scanning microscopy (CLSM), scanning electron microscopy (SEM), transmission electron microscopy (TEM), desktop microcomputed tomography, and synchrotron radiation-based CT (SR CT). Other techniques that are less commonly used are ptychographic X-ray CT, transmission X-ray microscopy (TXM) CT, serial-focused ion beam SEM (serial FIB SEM), and serial block-face SEM (SBF SEM). Different imaging techniques of the osteocyte lacunar network have been reviewed [33•, 34, 35, 36••, 37]. Table 1 briefly summarizes the pros and cons of the imaging methods of the osteocyte lacunar network.

**Table 1:** Imaging methods of osteocyte and lacunar network visualization

Techniques	2D/3D	Resolution	Sample preparation	Destructive	Penetration depth	Acquisition time	Field of view	Soft tissue contrast	Requirement of proper staining	Functional imaging through fluorescent labeling	References
Light microscopy (LM)	2D	Low (200 nm)	−	−	−	−	+	+	−	+	[38–43]
Confocal laser scanning microscopy (CLSM)	2D/3D	Low (200 nm)	−	−	−	−	+	+	−	+	[10•, 44•, 45–50]
Scanning electron microscopy (SEM)	2D	High (2 nm)	−	−	+	−	−	−	+	−	[51•, 52, 53]
Transmission electron microscopy (TEM)	2D	High (1 nm)	−	−	−	−	−	+/−	+	+	[54–56]
Desktop microcomputed tomography (desktop μCT)	3D	Low (> 100 nm)	+	+	+	−	−	−	+	−	[9•, 10•, 57–59]
Synchrotron radiation-based CT (SR-μCT)	3D	Low (> 50 nm)	+	+	+	−	−	−	+	−	[35, 60•, 61•, 62–68]

+ indicates the pros of the technique; − indicates the cons of the technique

*2D* 2 dimension, *3D* 3 dimension

Historically, histology was the method of choice to evaluate porosity. Although quantitative two-dimensional (2D)-histological and microscopic imaging techniques provide unique data on bone tissue dynamics, they cannot provide a complete visualization of bone microstructure as they are based on a limited number of 2D-sections. Additionally, 2D-methods typically overestimate bone microarchitecture because of preparation artifacts, and they are destructive in nature [69, 70]. These limitations might lead to misinterpretation. Therefore, a reliable and nondestructive method that allows to image at submicron resolution with a large field of view for a precise and accurate visualization and quantification and that avoids misinterpretation is needed. CT-based techniques are nondestructive and represent a 3D-methodology for characterization of biological tissues. The main advantage of 3D-imaging technique-based CT is that they provide nondestructive quantitative data without preparation of the sample. Afterwards, the sample can still be processed for (dynamic) histomorphometry or immunohistochemistry. Recently, we showed that desktop microCT allows an accurate and precise visualization and quantification of the osteocyte lacunar network in the bone [57].

A direct measurement of the 3D stresses and strains acting on the osteocytes is extremely challenging, if not impossible [71]. As an alternative, most efforts have tried to quantify these using computational models. Computational models have been based on the idealized LCN geometries, yet it has been shown that idealized models are not appropriate to evaluate lacunar strains [72]. Indeed, recent developments in finite element (FE) modeling of the LCN based on confocal laser scanning microscopy have demonstrated huge variations between the results obtained from idealized and more realistic models [73, 74]. Hence, an accurate quantification of the lacunar network is essential. Based on accurate and highly-detailed computational models representing the osteocyte lacunar network, fluid shear stresses at the cell level can be calculated using computational fluid dynamic (CFD) [75•, 76] and finite element (FE) models [71, 72]. The development in 3D-visualization of the osteocyte LCN allows FE analysis based on realistic models of osteocytes and their canalicular network [77, 78, 79].

## Osteocytes Modify Their Microenvironment, Leading to Alterations in Mechanotransduction

As argued in the previous paragraphs, morphological alterations in the LCN are likely to affect the ability of osteocytes to sense and respond to mechanical stimuli. Osteocytes do not undergo these modifications passively, but they are actively involved in shaping their microenvironment and play a key role in maintaining bone mineral homeostasis. Indeed, given the high number of osteocytes and the extent of the LCN [80], small changes in the dimensions of the LCN would effectively lead to alterations in mechanotransduction. Osteocytes can enlarge their lacunar volume by removing the bone from their perilacunar bone matrix in a process called osteocytic osteolysis or perilacunar remodeling. Conversely, they can promote perilacunar bone formation, thereby reducing the lacunar volume [81, 82•, 83, 84]. Probably, the best evidence for osteocytic osteolysis and perilacunar bone formation is that during lactation in mice, the volume of osteocyte lacunae is larger than the osteocyte lacunar volume in virgin and postweaned animals in both the cortical and trabecular bone [82•]. Furthermore, continuous administration of parathyroid hormone can cause osteocytic osteolysis in the cortex of rat tibia [84].

Modifications in the morphology and orientation of osteocytes and their lacunae could result from hormonal changes, as well as from changes in mechanical loading. The alignment and shape of the osteocytes and their lacunae have been shown to be related to the direction of the mechanical loading [10•, 85]. Vatsa et al. [10•] found more flattened and elongated osteocytes and lacunae in fibula loaded unidirectionally than in the calvarial bone, which is loaded in different directions. Sugawara et al. [85] demonstrated irregularly shaped osteocytes distributed in different directions in the femur of embryonic mice in the absence of mechanical loading, whereas the osteocytes in the femur of 6-week-old mice subjected to mechanical loading were more flat and spindle-shaped, and orientated parallel to the longitudinal axis of the bone. Furthermore, in neurectomized mice under little or no mechanical loading during growth, the osteocytes were round without any preferred orientation. The actin filaments in the osteocyte cytoskeleton distribute in the same direction as the mechanical loading [86]. Besides, the osteocyte morphology might vary in bone pathologies, i.e., the shape of osteocytes and their lacunae are significantly different in the tibia of individuals with osteoporosis, osteopenia, and osteoarthritis [9•]. Osteocyte lacunae in the bone from osteopenic persons are large and round, lacunae from osteopetrotic persons are small and discoid shaped, whereas lacunae from osteoarthritic persons are large and elongated [9•]. Furthermore, in the osteopetrotic bone, the osteocyte lacunae are less orientated to the loading direction in comparison with the orientation of osteocyte lacunae in the osteoarthritic and osteopenic bone [9•]. The variation in the shape and the alignment of osteocytes and their lacunae in different bone pathologies could reflect an adaptation to the different micromechanical environment with different matrix strain associated with differences in bone mineral density; however, conclusions about causal relations cannot be drawn based on observational data.

## Age-Related Changes in Bone Macro, Micro, and Nanostructure

With advancing age, a negative balance in bone remodeling results in decreased bone mass and alterations in the bone structure at the macroscale, microscale, and nanoscale, which may be associated with decreased bone mechanical properties [44•, 87•, 88], eventually leading to increased fracture risk with aging. In this section, we will report the aging-related changes in the bone architecture at different length scales, discuss the alterations in the mechanical properties as a consequence of bone structural changes, and evaluate the potential role of the osteocytes.

Aging is associated with changes in the geometrical macrostructure of both the trabecular and cortical bone. In the trabecular bone, aging is associated with a reduction in trabecular number, increased trabecular spacing, and unaffected or decreased trabecular thickness [89, 90]. In the cortical bone, aging causes endocortical resorption and formation on the periosteal surface, leading to cortical thinning and marrow cavity expansion.

The mechanical quality of the bone does not only depend on bone geometry at the macrolevel [91–95], but also on microporosities (including the LCN) [96, 97••, 98, 99]. More specifically, changes in the volume of the LCN due to osteocytic osteolysis have been shown to affect the local mechanical properties of the bone [87•, 100••]. In mice, alteration in the volume of the LCN during lactation reduced the elastic modulus of the bone tissue by 13%. These changes were only temporary as the elastic modulus returned to normal levels by 1 week postlactation [100••].

Alterations in the lacuna density likely play an important role in mechanical failure behavior of the bone. First, the LCN has been hypothesized to have a direct effect on bone fracture behavior either by acting as local stress concentrators that cause crack initiation [8, 31], or by dissipating energy and acting as barriers to slow down the propagation of microcracks [101]. Second, it has been suggested that a sufficient number of osteocytes is necessary for a proper bone repair [97••]. Reduced osteocyte density due to osteocyte apoptosis and the accumulation of mineral in lacunae with aging causes disturbs the bone remodeling process. More specifically, mineralized lacunae lead to decreased energy absorbing and dissipating capacities of the bone [102], and may cause the bone to become more brittle and susceptible to fragile fracture. A reduction in osteocyte number density can cause disturbance of canalicular fluid flow and decreased microdamage detection, resulting in impaired bone repair and decreased bone resistance to fracture [97••, 103–105]. In addition, the reduction in canalicular number could result in poor connectivity between osteocytes, and a hampered nutrition to osteocytes which can affect osteocyte mechanosensivity and ultimately bone quality and fracture resistance [106••].

With aging, conflicting results on changes in osteocyte lacunar number density (lacuna number per bone volume or bone area) have been reported (Table 2). A reduction in osteocyte lacunar number density with aging has been reported in the human cancellous bone [38–41, 45, 51••], human iliac crest cortical bone [60•], and murine femoral cortical bone [44•]. This reduction could result from osteocyte apoptosis and subsequent mineralization of the lacunar space, called micropetrosis [51••, 107]. In contrast, an increase in the osteocyte lacunar density with age has been found in the female vertebral cancellous bone [42], whereas no dependency on age was detected in the cortical bone from the femora of women [61•], and in rat tibia [58]. These discrepancies might be attributed to the species investigated, sex, the different span of aging, sample size, tissue type, as well as to differences in analytical techniques used.

**Table 2:** The effect of aging on osteocyte lacunar network parameters

Refs	Method	Species	Region of analysis	Sample size	Sex	Span	Lacuna density	Lacuna size	Lacuna sphericity
Mullender et al., 1996 [40]	Histomorphometry (2D)	Human	Trabecular bone of the iliac crest	24 male and 5 females	Female and male	30–91 years	Lower with aging ↓		
Mori et al., 1997 [39]	Histomorphometry (2D)	Human	Femoral head with and without femoral neck fractures	9 young and 12 old	Female	Young: 16–66 years Old: 73–88 years	Lower in old and fractured subjects. ↓		
Vashishth et al., 2000 [41]	Histomorphometry (2D)	Human	Femoral middiaphyseal cortical bone	16 male and 9 female	Female and male	Female: 28–63 years Male: 16–73 years	Lower with aging ↓		
Qiu et al., 2002 [45]	Confocal microscopy (2D)	Human	Transiliac bone	94	Female	20–73 years	Lower lacunae with aging ↓ Higher empty lacunae with aging ↑		
Vashishth et al., 2005 [42]	Histomorphometry (2D)	Human	Vertebral cancellous bone	35 male and 29 female	Female and male	Male: 36–96 years Female: 23–91 years	Higher with aging in females ↑		
Torres-Lagares et al., 2010 [38]	Histomorphometry (2D)	Human	Cancellous bone (coronal suture)	100	Female and male	16–79 years	Higher in females and lower with aging in both genders ↓		
Busse et al., 2010 [51••]	Backscattered scanning electron microscopy (2D)	Human	Femora	16 female and 16 male	Female and male	The range between the 1st and 9th decade.	Lower with aging ↓		
Carter et al., 2013 [61•]	Synchrotron radiation μCT (3D)	Human	Anterior blocks from the femoral shaft	30	Female	20–86 years	Unaffected with aging	Lower with aging	Smaller, rounded and more equant with aging
Jast et al., 2013 [58]	High-resolution microcomputed tomography (3D)	Sprague-Dawley rats	Tibiae	30	Female	3–72 weeks	Unaffected by age		
Lai et al., 2015 [44•]	Confocal laser scanning microscopy (3D)	Mice-B6	Right femora	15	Male	15–32 weeks	Lower with aging ↓		
Bach-Gansmo et al., 2016 [60•]	Synchrotron radiation μCT (3D)	Human	Iliac crest biopsies (5 cm × 5 cm)	46 female and 42 male	Female and male	Female: 18–96 years Male: 22–94 years	Lower with aging when pooling data from both sexes. ↓	Unaffected by age and sex.	

*2D* 2 dimension, *3D* 3 dimension

**Fig. 1:**
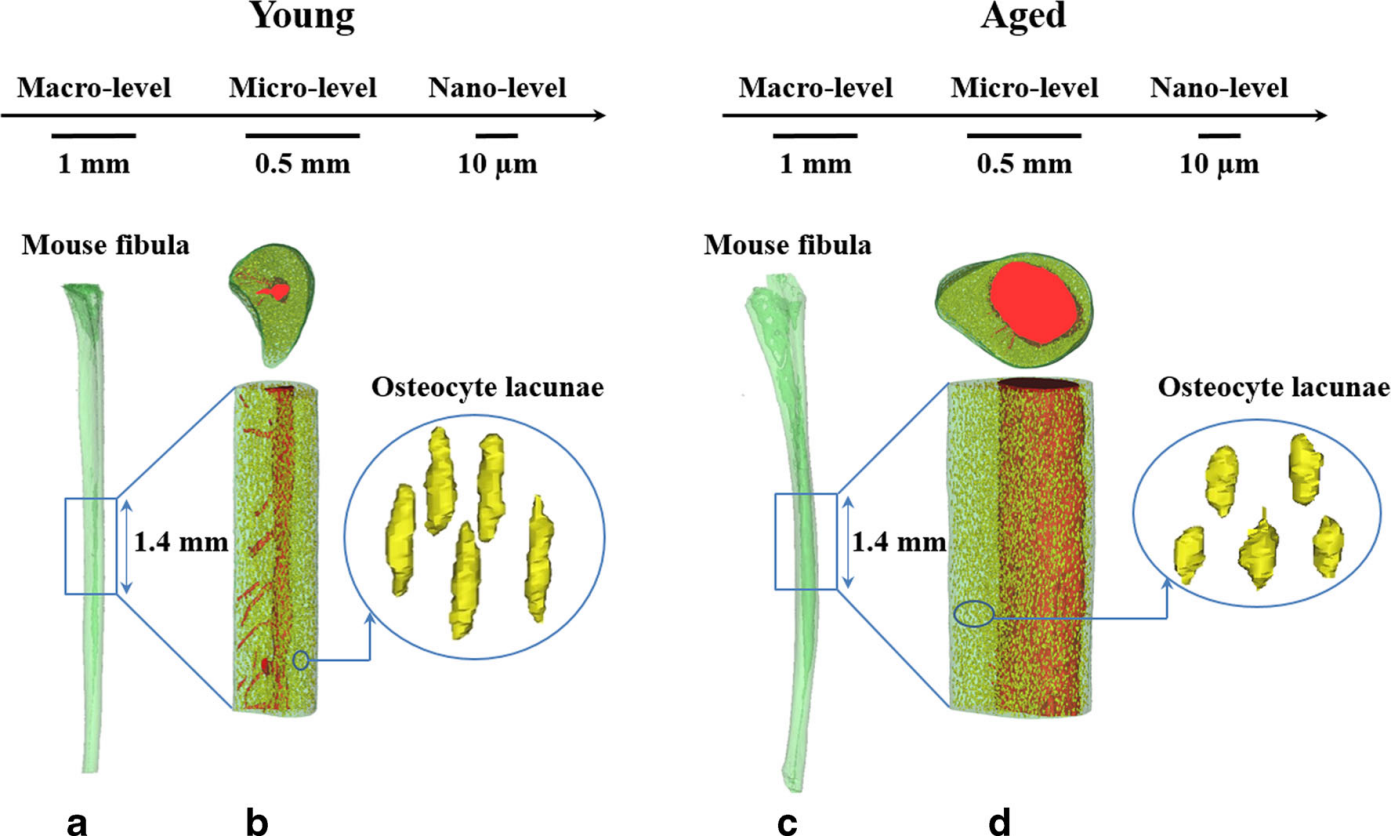
Aging alters bone structure at the macrolevel, microlevel, and nanolevel. Medullary area, mean periosteal perimeter, and mean endosteal perimeter are significantly larger for old mice compared with young ones. With advancing age, vascular canal density reduces. Furthermore, osteocyte lacunae become smaller and more spherical with increasing age. (A) 3D-rendering of a whole C57BL/6 female mouse fibula at young age (5-months) using microcomputed tomography (μCT) scans at 5-μm resolution. (B) 3D-rendering of osteocyte lacunae and vascular canal network together with medullary cavity at midfibula diaphysis at young age using μCT scans at 0.70-μm resolution. (C) 3D-rendering of a whole C57BL/6 female mouse fibula at old age (23-months) using μCT scans at 5-μm resolution. (D) 3D-rendering of osteocyte lacunae and the vascular canal network together with medullary cavity at midfibula diaphysis at old age using μCT scans at 0.70-μm resolution

In addition to osteocyte density, also osteocyte shape could affect bone mechanical behavior. As mentioned before, osteocyte lacunae have been hypothesized to be capable of amplifying local tissue strains around the osteocyte cell bodies. Hence, alterations in lacunar shape could affect the transition of strains to the direct osteocyte microenvironment [8]. The osteocytes will then experience a locally modified mechanical environment resulting in an adaptive response to mechanical loading [4••, 108]. With aging, changes in the morphology of osteocyte lacunae have been reported but again with conflicting data. Whereas the lacunae became smaller and more spherical in the human femora [61•], and in the fibula of C57BL/6 mice [109] (Fig. 1), no significant changes in lacuna volume were observed in the human iliac crest bone [60•], nor in the femora of C57BL/6 mice [44•]. Small lacunae with higher sphericity in the aged bones could result from ongoing mineralization of the lacunar space before complete micropetrosis occurs due to aging [61•, 107]. The lacunar shape changes are possibly reflected in changes in osteocyte shape, since the same morphology and alignment of osteocytes and their lacunae have been shown in situ using confocal laser scanning microscopy and nano-computed tomography [9•, 10•]. If osteocyte cell shape indeed changes with aging, this could relate to changes in mechanosensitivity. Osteocyte shape is dependent on cytoskeletal architecture [110, 111], which plays a key role in the osteocyte response to mechanical loading. Interestingly, round osteocytes are more mechanosensitive and require less mechanical force than flat ones in order to release nitric oxide, even though they are more compliant compared to flat osteocyte cells [11••]. Thus, based on this evidence, one can hypothesize that with aging, smaller and more spherical lacunae are related to smaller and more spherical osteocytes, which could be related to an altered capability to respond to mechanical loads leading to a change in the maintenance of bone mass and architecture.

A final aspect of the LCN to consider is the canaliculi. With aging, no dependence on age in canalicular number density was found in the cortical bone from the femora of women [44•]. On the other hand, a reduced number of canaliculi per lacuna has been found [106••, 43], and these were found to be smaller [52]. Theoretical considerations predict that a smaller amount of canaliculi per lacuna would give rise to lower strain levels around the osteocytes, leading to a reduced mechanosensitivity response of the osteocytes [31].

## Conclusion

In conclusion, aging is associated with changes in osteocyte lacuno-canalicular network (LCN) with respect to the shape and number density. Though extensive quantitative data is lacking, evidence exists that the osteocyte lacunae are becoming smaller and more spherical with aging. Additionally, in spite of conflicting results on age-related changes in osteocyte lacunar and canalicular number density, mostly, a reduction with aging has been reported. Since osteocytes can sense matrix strain directly via their cell bodies and the magnitude of the osteocyte signal is likely related to the number of osteocytes and their canaliculi contributing to the signal, the variations in osteocyte morphology and osteocyte number density may cause changes in mechanotransduction. This could be related to an altered capability to respond to mechanical loads leading to a change in the maintenance of bone mass and architecture with aging. Yet, whether the shape of the osteocyte lacuna can affect the bone mechanobiological response still needs confirmation. Considering the crucial role of osteocyte to maintain a healthy bone, a better understanding of the way osteocyte shape is related to its capability to direct bone formation and resorption may help to unravel whether changes in osteocytes and the LCN are related to the reduced bone adaptive response as seen with aging.
